# Perioperative Outcomes of Total Hip Arthroplasty in Trisomy 21 Patients in Comparison With a Control Population: Analysis From a Large National Sample of 367,894 Patients

**DOI:** 10.7759/cureus.68947

**Published:** 2024-09-08

**Authors:** Vishesh Khanna, Joshua Sun, Jonathan M Jose, Jacob P Scioscia, Varatharaj Mounasamy, Senthil Sambandam

**Affiliations:** 1 Orthopedics, Morriston Hospital, Swansea, GBR; 2 Orthopedics, University of Texas Southwestern Medical Center, Dallas, USA; 3 Orthopedics, Baylor College of Medicine, Houston, USA

**Keywords:** down's syndrome, morbidity, mortality, perioperative outcome, total hip athroplasty

## Abstract

Background: Literature on the outcomes of total hip arthroplasty (THA) has established the procedure as a gold standard for hip arthritis. However, postoperative outcomes after THA in specific conditions such as Down's syndrome (DS) have been sparsely described. This large database analysis of over 367,000 patients was aimed at evaluating the immediate postoperative results including morbidity and mortality rates after THA among DS patients and comparing these with a control population.

Methods: Data from the National Inpatient Sample (NIS) database Healthcare Cost and Utilization Project (HCUP) was reviewed retrospectively from 2016 to 2019 on THAs. Among 367,894 patients, 129 were identified with a diagnosis of DS. Complex primaries and revisions were excluded. Demographics, admission details, and perioperative variables including morbidity and mortality rates were compared between DS patients and controls.

Results: Patients with DS were younger than the control population (43.3 versus 65.9 years, p=0.002), had a greater preponderance of men, had a lower incidence of smoking and diabetes, and had a relatively higher incidence of non-elective THA. The former also had a longer mean length of stay (LoS) and higher mean costs to healthcare. Two patients with DS died after a THA, making the mortality rate 17-fold higher in DS patients. Higher rates of postoperative anemia (31.8% versus 19.6%, p<0.001), pneumonia (2.3% versus 0.3%), and pulmonary embolism (p=0.0.12) were seen in the DS group. Also seen in the DS group were higher risks of periprosthetic fractures (p=0.020) and periprosthetic joint infections (PJIs) (p=0.047).

Conclusions: Results from a total hip arthroplasty continue to positively transform the lives of patients with end-stage hip arthritis from varying etiologies. In the special cohort of Down's syndrome, a thorough discussion is essential with reference to the relatively higher morbidity and mortality in this group of patients. Documented conversations between patients and their families and healthcare providers should consist of detailed deliberations on the pros and cons of surgery and its potential impacts.

## Introduction

Research from the latter half of the previous century has identified the unique anatomical details of the hip in patients with Down's syndrome (DS). Shaw et al. [1] published findings from 100 patients with Down's syndrome, describing anatomical differences such as increased acetabular depth and anteversion, decreased abduction angle, and increased femoral anteversion. The combined incidence of dysplasia, dislocation, physeal slips, and osteonecrosis of the femoral head is as high as 8% [1]. There have been recent reports that have challenged the acetabular version and depths in DS patients [2]. These anatomical differences are purported to increase joint reactive forces across the hip joint, predisposing to degenerative arthritis of the hip, the incidence of which can range between 10% and 30% in patients with Down's syndrome [3].

Total hip arthroplasty (THA) in this scenario can be quite challenging in the background of increased tissue laxity and muscle hypotonia. Implant survival rates at a mean of 8.75 years have been reported to be over 90% [4]. Similar results have emerged from a broader analysis of nine studies including 321 patients with Down's syndrome, wherein a 7.5% revision rate at five years has been observed [2]. Midterm outcomes have shown an improvement in Harris Hip Scores from 37.9 to 89.2 at a mean of 5.8 years of follow-up [5]. Harbingers of surgical success have included consistent and meticulous techniques along with judicious use of constrained implants [6].

Despite reports of success and improvement of hip scores, revision rates and complication rates in patients with trisomy 21 are two- and threefold, respectively [7]. Further reports suggest a high incidence of perioperative complications and length of stay (LoS) among patients with Down's syndrome [8]. The need for a larger and wider national analysis of the comparative outcomes between the former and a control population has been highlighted in the literature. Our aim was to identify immediate postoperative mortality (primary outcome) and morbidity and complications (secondary outcomes) in trisomy 21 patients undergoing THA from a large database of over 367,000 patients. We compared these outcomes to controls from the same national cohort. We hypothesized that significant differences would not exist in outcomes between the two groups.

## Materials and methods

Data source

For the purpose of this study, data from the National Inpatient Sample (NIS) database Healthcare Cost and Utilization Project (HCUP) was utilized [9]. The latter is one of the biggest data banks in the USA, accounting for almost 20% of overall annual hospital admissions involving joint replacement surgeries. The database comprises over seven million hospital stays per year and is checked using quality assessment tools. The data is also compared with standardized values by an independent contractor [9]. The International Classification of Diseases 10th revision codes (ICD-10-CM/PCS) are utilized by the NIS. Codes for DS patients included were Q900, Q901, Q902, and Q909. Details for other codes are enclosed in the Appendices.

In the current paper, prospectively recorded data on the NIS was reviewed retrospectively from 2016 to 2019. Patients undergoing THA and those with trisomy 21 were identified from this cohort while maintaining strict confidentiality with patient identifiers. Complex and revision surgeries were not included in the analysis. Among the 367,894 patients who had undergone THA, 129 patients had a diagnosis of trisomy 21 (0.03%).

Data collection and statistical analysis

Demographic parameters (age, gender, and race), comorbidities (diabetes, tobacco use, and obesity), admission details (elective versus non-elective), LoS, total charges, and mortality rates were all included and compared between the Down's syndrome (DS) and control group. Medical and surgical perioperative complications (renal failure, myocardial infarction, blood loss, pneumonia, thromboembolism, fractures, wound complications, and infections) were also analyzed. Calculations were computed using SPSS version 27.0 8 (IBM Corp., Armonk, NY) with descriptive statistics for demographic data and analytical statistics for perioperative factors in THA in DS patients versus controls. T-tests were employed to determine the significance of LoS, age, and costs. To determine the significance of binomial variables, chi-square tests were employed, and for values <5, Fisher's exact test was used. The Pearson chi-square tests were utilized for those with values ≥ 5 (statistical significance: p<0.005).

## Results

Among 367,894 patients undergoing THAs, 129 had Down's syndrome (DS group). Table 1 shows that the mean ages in the DS group and controls were 43.3 and 65.9 years (p=0.002), respectively. Males had a greater demographic presence among the DS group (58.1%), while the control group had a slight female preponderance of 56% (p=0.001). Racial differences were not different between the two groups. A higher incidence of smoking and diabetes was observed in the control population. Also, a relatively higher incidence of elective THA was seen in the control group of patients versus the DS group (91.3% versus 79.8%, respectively).

**Table 1:** Demographic data of DS patients versus controls DS: Down's syndrome

Variable	DS group (n=129)	Controls (n=359,993)	p
Age at admission (years)	43.3	65.9	0.002
Gender (female)	54 (41.9%)	205,688 (55.9%)	0.001
Gender (male)	75 (58.1%)	162,039 (44.1%)	
Elective	103 (79.8%)	335,450 (91.3%)	<0.001
Non-elective	26 (20.2%)	31,808 (8.7%)	
Diabetes without complications	6 (4.7%)	36,822 (10%)	0.022
Diabetes with complications	0 (0%)	713 (0.2%)	0.779
Tobacco-related disorder	0 (0%)	63,708 (17.3%)	<0.001
Obesity	29 (22.5%)	79, 890 (21.7%)	0.459
Race	DS group (n=122)	Controls (n=354,131)	-
White	100 (81.9%)	303,002 (85.6%)	-
Black	9 (7.4%)	27,553 (7.8%)	-
Hispanic	4 (3.3%)	13,030 (3.7%)	-
Asian/Pacific Islander	3 (2.5%)	3,407 (1%)	-
Native American	0 (0%)	1,122 (0.3%)	-
Other	6 (4.9%)	6,017 (1.7%)	-

Longer mean LoS was seen in the DS group (4.3 days) vis-a-vis the control group after a THA (2.3 days, p<0.001). Additionally, mean costs to healthcare from the former group were significantly higher (p<0.001) as shown in Table 2. There were two in-hospital deaths among the 129 patients in the DS group, which saw a 17-fold higher mortality rate when compared with controls (1.6% versus 0.09%, respectively) (Table 2) (Pearson chi-square: p<0.001, Fisher's exact test: p=0.006).

**Table 2:** LoS, healthcare costs, and mortality in the DS group versus the control group LoS: length of stay, DS: Down's syndrome

Variable	DS group (n=129)	Controls (n=367,761)	p
Mean length of stay (days)	4.3	2.3	<0.001
Total charges ($)	$93,003.7	$66,870.8	<0.001
Variable	DS group (n=129)	Controls (n=367,627)	p
Died during hospitalization	2 (1.6%)	330 (0.09%)	0.006

Although a relatively greater incidence of postoperative anemia was seen in the DS group (31.8% versus 19.6%, p<0.001), the incidence of blood transfusions was similar in both groups (3.9% versus 3.5%, p=0.808) as seen in Table 3. A higher incidence of pneumonia (2.3% versus 0.3%) and pulmonary embolism (p=0.012) were seen in the DS group, while similar incidences of acute renal failure (3.1% versus 2.5%), myocardial infarction (p=1.000), and deep vein thrombosis (p=1.000) were seen in the DS and control group.

**Table 3:** Systemic complications among DS patients and controls after a THA **Could not be calculated since the value was 0 ***HCUP user agreement does not allow publishing values between 1 and 10 DS: Down's syndrome, THA: total hip arthroplasty, HCUP: Healthcare Cost and Utilization Project, CI: confidence interval, PE: pulmonary embolism, DVT: deep vein thrombosis

Complication	DS (n=129)	Controls (n=367,894)	Odds ratio	95% CI	p
Acute renal failure	*** (3.1%)	9,126 (2.5%)	0.795	(0.294, 2.152)	<0.566
Myocardial infarction	** (0%)	142 (0.04%)	1.000	(1.000,/ 1.000)	1.000
Anemia	41 (31.8%)	71,930 (19.6%)	0.522	(0.360,/ 0.756)	<0.001
Blood transfusion	*** (3.9%)	12,897 (3.5%)	0.901	(0.369,/ 2.203)	0.808
Pneumonia	*** (2.3%)	970 (0.3%)	0.111	(0.036,/ 0.349)	0.005
PE	*** (1.6%)	473 (0.1%)	0.082	(0.020, 0.331)	0.012
DVT	** (0%)	562 (0.2%)	-	-	-

Increased incidence of periprosthetic fractures was seen in the DS group (p=0.020), while the risk of mechanical complications, dislocations, wound dehiscence, and surgical site infections did not seem to differ between the two groups (Table 4). However, periprosthetic joint infection (PJI) rates were three times higher in the DS group (p=0.047).

**Table 4:** Local complications among the DS group versus the control group after a THA **Could not be calculated since the value was 0 ***HCUP user agreement does not allow publishing values between 1 and 10 SSI: surgical site infection, PJI: periprosthetic joint infection, DS: Down's syndrome, THA: total hip arthroplasty, HCUP: Healthcare Cost and Utilization Project, CI: confidence interval

Complication	DS (n=129)	Controls (n=367,894)	Odds ratio	95% CI	p
Periprosthetic fracture	*** (3.9%)	4,420 (1.2%)	0.302	(0.124,/ 0.738)	0.020
Periprosthetic dislocation	*** (0.8%)	5,150 (1.4%)	1.818	(0.254,/ 13.000)	0.459
Periprosthetic mechanical complications	*** (1.6%)	2,854 (0.8%)	0.497	(0.123,/ 2.007)	0.265
Wound dehiscence	*** (0.8%)	304 (0.08%)	0.107	(0.015,/ 0.762)	0.102
Superficial SSI	0 (0%)	43 (0.01%)	**	**	0.985
Deep SSI	0 (0%)	30 (0.01%)	**	**	0.990
PJI	*** (3.1%)	3,827 (1%)	0.329	(0.122,/ 0.889)	<0.047

A greater number of DS patients needed supported discharge, and only one in four patients from the group were routinely discharged home after THA (Table 5). Most patients from this group required rehabilitation at another facility.

**Table 5:** Discharge destinations in the DS group versus the control group ***HCUP user agreement does not allow publishing values between 1 and 10 DS: Down's syndrome, HCUP: Healthcare Cost and Utilization Project, HHC: Home Health Care, AMA: against medical advice

Variable	DS (n=129)	Controls (n= 367,627)
Routine	32 (24.8%)	143,206 (38.9%)
Short-term hospital stays	*** (1.6%)	868 (0.2%)
Another facility	51 (39.5%)	67,447 (18.3%)
HHC	42 (32.6%)	155,522 (42.3%)
AMA	0 (0%)	253 (0.07%)
Died	***	330 (0.09%)

## Discussion

The results from this large study reveal that the cohort of patients undergoing a THA with trisomy 21 tend to have a younger mean age at surgery, are more often male, and have a lower incidence of smoking. The immediate outcomes in Down's syndrome patients demonstrate increased length of stay and total healthcare cost. Although THA is regarded as a safe surgery in the modern context, patients with Down's syndrome experienced a 17 times increase in postoperative mortality (1.6% versus 0.09%). Therefore, we reject the null hypothesis.

The study represents a perioperative assessment of one of the largest pools of data in patients with trisomy 21 who have undergone THA. With the continued rise in both quality of care and life expectancy for patients with Down's syndrome, there comes a higher demand for THA [4]. The incidence of THA in Down's syndrome patients among the general population is 0.04%, which is very similar to previously reported data from a large national analysis, where 241 trisomy 21 patients underwent a THR between 1998 and 2010 among 543,085 patients [8]. Younger mean ages were reported in the DS group versus controls (43.3 versus 65.9 years, p=0.002), and the present study has similar findings (42.2 versus 65.1 years, p=0.001) [8]. The inclusion of data from a 10-year period in a fast-paced subspecialty of orthopedics can potentially induce bias and comes with its limitations. Emerging trends in joint replacement surgery can influence outcomes and skew data for analysis. It is for this reason that a shorter, three-year period was chosen in the present paper.

In an era of fast-track arthroplasty, over 90% of patients are discharged under four days postoperatively [10], and the controlled comparative mean LoS in patients with DS has shown to be nearly twice as long in the present paper (4.3 versus 2.3 days, p<0.001). This finding indicates a decrease in the mean of 4.8 days for DS patients from data between the years 1998 and 2010 and could reflect a pattern of changing practices and/or a move toward day-case arthroplasty [8]. Expectedly, THA has increased costs associated for DS patients ($93,003.7) versus controls ($66,870.8). This data point was previously noted to have a smaller gap in a decade-old database ($40,216 versus $39,148) [8]. While it is difficult to attribute LoS in isolation to this increase, a further detailed analysis was beyond the scope of this paper. A potentially cost-saving strategy for this could be operating in specialized orthopedic units vis-a-vis general non-specialized units as highlighted by Batsis et al. [11].

In a 12-year analysis of NIS data between 1998 and 2010, Boylan et al. [8] demonstrated higher complication rates in DS patients when compared with matched controls. The significant specific risks associated with DS included pneumonia (2.9% versus 0.3%, p=0.001), urinary tract infection (6.6% versus 1.4%, p<0.001), and bleeding (2.1% versus 0.4%; p=0.027) [8]. In our results, medical complications noted significantly more frequently in the DS group included postoperative anemia, pneumonia, and pulmonary embolism (p=0.012). Both immunological and non-immune-mediated mechanisms have been cited in the published literature as explanations for the relatively higher risk of postoperative infections in DS patients [12]. These figures agree with statistics from a recent study on 154 patients with DS receiving a THA and a matched analysis with 1,532 controls. The authors also included total knee arthroplasty patients in their analysis and noted higher postoperative and 90-day complications including sepsis, minor events, urinary infections, acute kidney injury, and respiratory complications including pneumonia. The rates of revision were not different at two years postoperatively. The study, however, did not specifically evaluate the immediate postoperative, pre-discharge outcomes in these patients [13].

Analysis of 14 DS patients who underwent THA found two intraoperative fractures [5]. Although the aforementioned study lacks a comparative analysis with controls, it reflects a high incidence of periprosthetic fractures in DS patients akin to that reflected in the present paper versus controls (p=0.020). We did not observe any similar trends of increased mechanical complications when comparing DS patients with controls. The incidence of periprosthetic joint infection being higher in DS patients is potentially explained by a compromised immune system and increased predisposition to infection. This has been reported differently in literature with no apparent increase in the incidence of PJI at 90 days and two years postoperatively [14]. A higher mortality was also observed in the present analysis among the DS group versus the control group (1.6% versus 0.09%, p<0.001), which has not been previously observed (p=0.25) [8]. As these figures arise from an extension of the same NIS database, a future study could potentially explore the exact causes of this. Understandably, DS patients required greater rehabilitation needs and a higher transfer to caring facilities rather than home as an initial destination.

We acknowledge that, in our study, the population groups significantly differed in their sizes. The rarity of the condition and overall paucity of data on patients with trisomy 21 undergoing a THA made the analysis of each patient important despite the difference in the size of the two groups. Similar variability was noted in age and gender distribution between the two groups, which could have potentially affected interpretations of outcomes. Although a matched study could have addressed this disparity, it would have had to be spread across several years to gather data of similar magnitude. This in turn would have discounted evolving practices, making them less consistent as a three-year period has reflected in the present study.

The exact details for mortality in both groups are very difficult to draw. We were able to categorize the differences in specific complications, including cardiac and pulmonary events, which were possibly linked to the difference in mortality between the two groups. Another proposed reason could be a relatively higher incidence of non-elective THA in the DS group. Results from a French study in 2015 have shown that among 319,804 patients, mortality rates for THA for trauma were 3.42% when compared to elective patients (0.18%) [15]. These were similar in our analysis.

Long-term and follow-up information were not studied in the present research, and statistical analysis was limited by the use of simple intergroup comparisons rather than multivariate analysis. This was primarily restricted by logistical barriers to accommodate follow-ups and remains an area of further exploration. Also, a matched analysis was not performed for this rare diagnosis and an even rarer procedure on this cohort of patients. This would have offered advantages but would carry risks of inducing selection and sparse-data bias [16].

The strengths of this paper are illustrated in the volume of data analyzed and translatable results to add to modern evidence-based medicine. Only one study (Boylan et al. [8]) has been published on a larger sample size; however, this data is now a decade old. Rapidly evolving trends necessitate a continual appraisal of up-to-date data and weighing them with current practices.

The aggregation of data from multiple centers could have, understandably, led to variability, especially regarding different surgical practices nationally. These could include choice of approach and prostheses among others. Although this was beyond the scope of the current paper, it remains an interesting prospect to explore for future studies.

## Conclusions

To conclude, total hip arthroplasty is a life-transforming surgical intervention with an increasingly reliable record in patients with end-stage hip arthritis from various etiologies. In a separate cohort of trisomy 21 patients with hip pathologies, special care needs to be taken and given while working up patients who cannot manage with non-operative interventions. A sensitive and elaborate discussion is requisite with reference to the relatively higher morbidity and mortality in this group of patients, which should preoperatively take place between the surgeon, patient, and their healthcare providers.

## Appendices

The details of other codes used in this study are shown in Table 6.

**Table 6:** ICD codes used ICD: International Classification of Diseases, DVT: deep vein thrombosis, SSI: surgical site infection

Down's syndrome	Total hip replacement codes	Obese codes	Morbidly obese codes	Comorbidities codes	Medical complications codes	Surgical complications codes
Q900 Q901 Q902 Q909	0SR9019 0SR901A 0SR901Z 0SR9029 0SR902A 0SR902Z 0SR9039 0SR903A 0SR903Z 0SR9049 0SR904A 0SR904Z 0SR9069 0SR906A 0SR906Z 0SR907Z 0SR90EZ 0SR90J9 0SR90JA 0SR90JZ 0SR90KZ 0SRB019 0SRB01A 0SRB01Z 0SRB029 0SRB02A 0SRB02Z 0SRB039 0SRB03A 0SRB03Z 0SRB049 0SRB04A 0SRB04Z 0SRB069 0SRB06A 0SRB06Z 0SRB07Z 0SRB0EZ 0SRB0J9 0SRB0JA 0SRB0JZ 0SRB0KZ	E660 E6601 E6609 E661 E662 E668 E669 Z6830 Z6831 Z6832 Z6833 Z6834 Z6835 Z6836 Z6837 Z6838 Z6839	Z6841 Z6842 Z6843 Z6844 Z6845	Diabetes without complications: E119, diabetes with complications: E1169, tobacco-related disorder: Z87891	Acute renal failure: N170, N171, N172, N178, N179; myocardial infarction: I2101, I2102, I2111, I2113, I12114, I12119, I2121, I12129, I21A1; blood loss anemia: D62; pneumonia: J189, J159, J22; blood transfusion: 30233N1; pulmonary embolism: I2602, I2609, I2692, I2699; DVT: I82401, I82402, I82403, I82409, I82411, I82412, I82413, I82419, I82421, I82422, I82423, I82429, I82431, I82432, I82433, I82439, I82441, I82442, I82443, I82449, I82491, I82492, I82493, I82499, I824Y1, I824Y2, I824Y3, I824Y9, I824Z1, I824Z2, I824Z3, I824Z4	Periprosthetic fracture: T84010A, T84011A, T84012A, T84013A, T84018A, T84019A, M9665, M96661, M96662, M96669, M96671, M96672, M96679, M9669, M9701XA, M9702XA, M9711XA, M9712XA; periprosthetic dislocation: T84020A, T84021A, T84022A, T84023A, T84028A, T84029A; periprosthetic fracture: T84090A, T84091A, T84092A, T84093A, T84098A, T84099A; periprosthetic infection: T8450XA, T8451XA, T8452XA, T8453XA, T8454XA, T8459XA; deep SSI: T8142XA; wound dehiscence: T8130XA, T8131XA, T8132XA
